# Thalamic Structural Connectivity Abnormalities in Minimal Hepatic Encephalopathy

**DOI:** 10.3389/fnana.2021.592772

**Published:** 2021-02-25

**Authors:** Hua-Jun Chen, Xiao-Hong Zhang, Jia-Yan Shi, Shao-Fan Jiang, Yi-Fan Sun, Ling Zhang, Dan Li, Rong Chen

**Affiliations:** ^1^Department of Radiology, Fujian Medical University Union Hospital, Fuzhou, China; ^2^Department of Radiology, The First Affiliated Hospital of Nanjing Medical University, Nanjing, China; ^3^Department of Gastroenterology and Fujian Institute of Digestive Disease, Fujian Medical University Union Hospital, Fuzhou, China; ^4^Department of Diagnostic Radiology and Nuclear Medicine, University of Maryland School of Medicine, Baltimore, MD, United States

**Keywords:** minimal hepatic encephalopathy, thalamus, probabilistic tractography, cognition, diffusion tensor imaging

## Abstract

**Background and Aims:** Numerous studies have demonstrated thalamus-related structural, functional, and metabolic abnormalities in minimal hepatic encephalopathy (MHE). We conducted the first study to investigate thalamic structural connectivity alterations in MHE.

**Methods:**

Diffusion tensor imaging (DTI)-based probabilistic tractography was employed to determine the structural linkage between the thalamus and cortical/subcortical regions in 52 cirrhotic patients [22 with MHE; 30 without MHE (NHE)] and 30 controls. We measured these thalamic connections, which included connectivity strength (CS), fractional anisotropy (FA), mean diffusivity (MD), axial diffusivity (AD), and radial diffusivity (RD), and then compared these among the three groups. Neurocognitive assessment was also performed. Correlation analysis was conducted to investigate the relationship between neurocognitive performance and the above measurements. Classification analysis was performed to determine whether thalamic connection measurements can distinguish MHE from NHE.

**Results:**

The probabilistic tractography revealed thalamic structural connections, which were disrupted in cirrhotic patients (as reflected by a decrease in CS/FA and an increase in MD/AD/RD). Abnormal thalamic connections primarily involved the prefrontal cortex, sensorimotor cortex, parietal cortex, medial temporal cortex and hippocampus, and striatum. Thalamic connectivity abnormalities deteriorated from NHE to MHE, and they were correlated with patients’ neurocognitive performance. The moderate classification accuracy was obtained using CS and MD as discriminating indexes.

**Conclusion:**

Our results demonstrated the altered thalamic structural connectivity involving both cortical and subcortical regions in MHE, which could be regarded as representative of MHE-related widespread impairments in white matter pathways. The disturbed thalamic connectivity may underlie the mechanism of cognitive deficits in MHE and may potentially be utilized as a biomarker for diagnosing MHE and in monitoring disease progression. In addition to thalamic–cortical/subcortical connections, further studies are recommended to explore the structural alterations in other white matter pathways in MHE.

## Introduction

Minimal hepatic encephalopathy (MHE) is the most frequently observed neurocognitive complication of cirrhosis, and it is characterized by a range of cognitive impairments, including a reduction in psychomotor speed, poor attention ability, reduced memory function, disrupted executive performance, and abnormal response control (Vilstrup et al., 2014). The morbidity of MHE in cirrhotic patients is up to 60% (Bajaj et al., 2009; Vilstrup et al., 2014). MHE impairs health-related quality of life and daily functioning (working disability and impaired driving skill), and it is associated with a higher risk of progression to overt hepatic encephalopathy (OHE), which often has an even poorer prognosis (Prasad et al., 2007; Bajaj et al., 2009; Vilstrup et al., 2014). Therefore, a consensus has been reached that patients with MHE should be treated, which may improve their cognitive performance and, subsequently, quality of life (Prasad et al., 2007). Despite the large attention delegated to MHE, the neuropathological mechanism underlying this disease remains unclear.

The thalamus is a critical pathological node of MHE. An increasing number of studies have demonstrated that MHE is associated with the disturbance of the basal ganglia–thalamus–cortical pathway (Zhang et al., 2012, 2014). The thalamus is the pivot node of this circuit; it integrates incoming information and transmits these to relevant cortical regions, as well as modulates cortical activity and supports the cortico-cortical communication (Goldstone et al., 2018). Abnormality of the thalamus can cause a range of cerebral dysfunctions, such as selective attention deficit, memory decline, executive dysfunction, and inhibitory control disability (Xia et al., 2012; Serra et al., 2014), all of which have been reported in MHE. In fact, histopathologically, neuronal cell death and myelinolysis involving the thalamus have been reported in hepatic encephalopathy (HE) (Soffer et al., 1995; Butterworth, 2007). In addition, neuroimaging studies have elucidated thalamus-associated structural, functional, and metabolic abnormalities in MHE. Structural magnetic resonance imaging studies have reported a volume reduction in the thalamus of cirrhotic patients with or without MHE (Montoliu et al., 2012). Moreover, functional abnormalities of the thalamus have also been observed in MHE. For example, a voxel-based functional correlation study demonstrated that cirrhotic patients show decreased functional connectivity strength (CS) in the thalamus (Chen et al., 2015). Also, a functional network analysis indicated disruptions of the thalamic functional connectivity with cortical/subcortical areas in MHE (Qi et al., 2013), which is associated with levels of blood ammonia and patient neurocognitive performance. Furthermore, in terms of thalamic metabolism, a positron emission tomography (PET)–computed tomography study (Lockwood et al., 1991) has revealed altered cerebral glucose metabolism in MHE patients.

Diffusion-based tractography has enabled us to map the thalamic structural network *in vivo*, and it shows good agreement with those found in functional and histological studies and is reproducible among subjects, using a powerful probabilistic approach (Behrens et al., 2003; Zhang et al., 2010). In contrast to the conventional streamline tractography that generates reliable fiber tracking results only in the areas with high anisotropy, this probabilistic method is able to map the fiber connectivity between brain regions and can trace pathways into the cortical and subcortical gray matter (Ciccarelli et al., 2006; Behrens et al., 2007). In fact, using probabilistic tractography, researchers have shown abnormalities of thalamic structural connectivity in attention deficit/hyperactivity disorder, schizophrenia, and other neuropsychological diseases (Marenco et al., 2012; Xia et al., 2012). A previous study has demonstrated the disruption of thalamic functional connectivity in MHE (Qi et al., 2013), prompting us to infer that the underlying alteration of thalamic structural connectivity occurs in MHE. Thus, we aimed to apply probabilistic tractography to investigate thalamic fiber pathway alterations in MHE.

## Experimental Procedures

### Participants

This study was approved by the ethics committee of Fujian Medical University Union Hospital and The First Affiliated Hospital of Nanjing Medical University, China. Each subject gave his/her written informed consent to be part of this study. A total of 52 cirrhotic patients [22 with MHE; 30 without MHE (NHE)] as well as 30 healthy control (HC) subjects were enrolled in this study. Patient demographic and clinical information is presented in Table 1. No significant differences in gender, age, or educational level were detected among the three groups. MHE was diagnosed based on the Psychometric Hepatic Encephalopathy Score (PHES) examination, which consisted of five subtests, i.e., line tracing test, digit symbol test, serial dotting test, and number connection tests A and B. The details on the PHES tests are described elsewhere (Chen et al., 2015). The patient with PHES ≤−5 was diagnosed as having MHE.

**TABLE 1:** Demographics and clinical characteristics of the study participants.

	HC (*n* = 30)	NHE patients (*n* = 30)	MHE patients (*n* = 22)	*P* value (ANOVA)
Age (years)	50.7 ± 7.2	51.9 ± 9.9	51.3 ± 9.0	0.87
Sex (males/females)	24/6	25/5	18/4	0.94 (χ^2^-test)
Education level (years)	8.8 ± 2.8	8.2 ± 3.2	8.9 ± 2.7	0.60
Etiology of cirrhosis (HBV/alcoholism/HBV + alcoholism/others)	–	21/4/2/3	14/4/2/2	–
Child–Pugh stage (A/B/C)	–	20/9/1	4/12/6	–
Final PHES	0.7 ± 1.6	−0.6 ± 2.2 ^†^	−8.3 ± 3.1*, ^#^	<0.001
Number connection test A (s)	33.9 ± 8.9	39.2 ± 10.8	57.4 ± 17.3*, ^#^	<0.001
Number connection test B (s)	53.0 ± 18.4	74.1 ± 26.7 ^†^	134.1 ± 62.1*, ^#^	<0.001
Serial dotting test (s)	39.1 ± 6.7	46.5 ± 9.3^†^	65.3 ± 18.5*, ^#^	<0.001
Digit symbol test (raw score)	49.1 ± 13.4	41.9 ± 12.1^†^	26.9 ± 8.5*, ^#^	<0.001
Line tracing test (raw score)	112.3 ± 21.8	144.4 ± 37.3^†^	192.3 ± 47.3*, ^#^	<0.001

MHE, minimal hepatic encephalopathy; NHE, patients without MHE; PHES, Psychometric Hepatic Encephalopathy Score; HC, healthy control. The symbols *, ^†^, and ^#^ indicate significant neurological performance differences in MHE vs. HC, NHE vs. HC, and MHE vs. NHE, respectively.

Patient exclusion criteria were as follows: (1) a current diagnosis of OHE or other neuropsychiatric disorder, (2) undergoing treatment using psychotropic medications, (3) diagnosed with uncontrollable endocrine or metabolic disorder (e.g., thyroid dysfunction), (4) alcohol abuse within the past 6 months prior to the study, and (5) other MRI contraindications.

### MRI Data

Imaging data were obtained using a 3.0 T MRI scanner (Siemens, Verio, Germany). We applied a single-shot echo-planar imaging sequence to collect diffusion tensor imaging (DTI) data using the following parameters: repetition time (TR) = 10,000 ms, echo time (TE) = 95 ms, slice thickness = 2 mm, slices = 70, field of view (FOV) = 256 mm × 256 mm, matrix = 128 × 128, orientation = axial, 30 non-linear diffusion-weighted gradient directions with *b* = 1,000 s/mm^2^, and one additional image without diffusion weighting (i.e., *b* = 0 s/mm^2^). Three-dimensional T1-weighted sagittal images of magnetization-prepared rapid gradient echo (MPRAGE) were gathered using the following settings: TR = 1.9 ms, TE = 2.48 ms, FOV = 256 mm × 256 mm, matrix = 256 × 256, flip angle = 9°, slice thickness = 1.0 mm, 176 slices.

### Fiber Tracking

**FIGURE 1:**
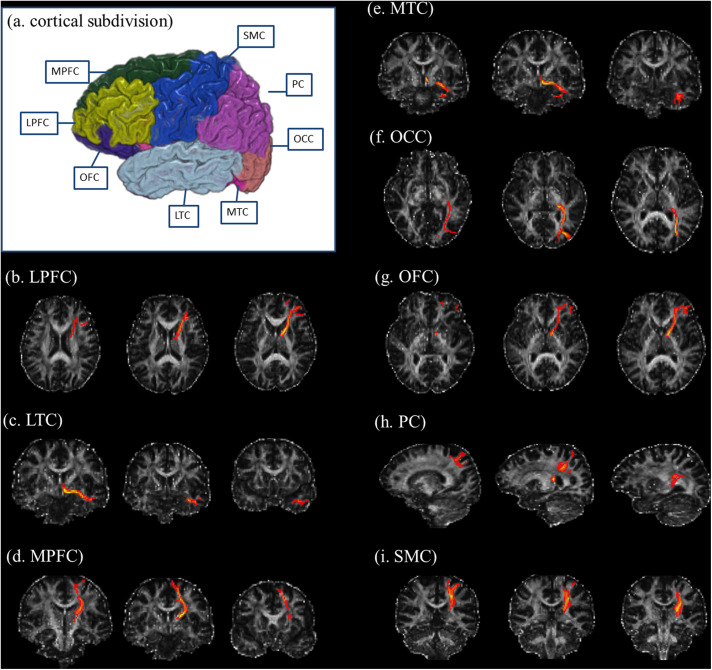
Illustration of cortical subdivisions and their connections with the thalamus. **(a)** After the FreeSurfer parcellations, the subsequent combination of brain regions was performed to generate the cortical subdivisions. The cortical subdivisions of the right hemisphere in one healthy volunteer are shown for visualization. Panels **(b–i)** illustrated the fiber tractography for thalamic connections with the LPFC, LTC, MPFC, MTC, OCC, OFC, PC, and SMC, respectively. LPFC, lateral prefrontal cortex (including the pars triangularis, frontal pole, rostral middle frontal gyrus, and pars opercularis); LTC, lateral temporal cortex (including the transverse temporal cortex, superior temporal gyrus, banks of the superior temporal sulcus, inferior temporal gyrus, middle temporal gyrus, and temporal pole); MPFC, medial prefrontal cortex (including the caudal anterior cingulate, rostral anterior cingulate, and superior frontal gyrus); MTC, medial temporal cortex (including the entorhinal cortex, parahippocampal gyrus, and fusiform gyrus); OCC, occipital cortex (including the pericalcarine cortex, lingual gyrus, lateral occipital cortex, and cuneus cortex); OFC, orbitofrontal cortex (including the pars orbitalis, medial orbitofrontal cortex, and lateral orbitofrontal cortex); PC, parietal cortex (including the inferior parietal cortex, supramarginal gyrus, precuneus cortex, posterior cingulate cortex, isthmus cingulate, and superior parietal cortex); SMC, sensorimotor cortex (including the precentral gyrus, caudal middle frontal gyrus, postcentral gyrus, and paracentral lobule).

We used FreeSurfer software^1^ to generate seed regions for DTI-based fiber tracking. Based on the T1-weighted images, FreeSurfer generated a parcellation with 80 regions (40 regions in each hemisphere, see^2^). Following the parcellation by FreeSurfer, we constructed a customized atlas, which included the thalamus, caudate, putamen, pallidum, hippocampus, amygdala, and eight cortical subdivisions in each hemisphere, according to previous DTI studies (Marenco et al., 2012; Xia et al., 2012; Fan et al., 2014). The eight cortical subdivisions were generated by a subsequent combination of brain cortical regions after the FreeSurfer parcellation (Marenco et al., 2012; Fan et al., 2014). They were the orbitofrontal cortex (OFC, including the pars orbitalis, medial orbitofrontal cortex, and lateral orbitofrontal cortex), the medial prefrontal cortex (MPFC, including the caudal anterior cingulate, rostral anterior cingulate, and superior frontal gyrus), the lateral prefrontal cortex (LPFC, including the pars triangularis, frontal pole, rostral middle frontal gyrus, and pars opercularis), the sensorimotor cortex (SMC, including the precentral gyrus, caudal middle frontal gyrus, postcentral gyrus, and paracentral lobule), the parietal cortex (PC, including the inferior parietal cortex, supramarginal gyrus, precuneus cortex, posterior cingulate cortex, isthmus cingulate, and superior parietal cortex), the medial temporal cortex (MTC, including the entorhinal cortex, parahippocampal gyrus, and fusiform gyrus), the lateral temporal cortex (LTC, including the transverse temporal cortex, superior temporal gyrus, banks of the superior temporal sulcus, inferior temporal gyrus, middle temporal gyrus, and temporal pole), and the occipital cortex (OCC, including the pericalcarine cortex, lingual gyrus, lateral occipital cortex, and cuneus cortex) (Figure 1).

DTI images are processed by an FSL-based pipeline^3^. FSL corrected raw DTI data for head movement and eddy-current distortion and then fitted a diffusion tensor model for each voxel. For each subject, the labeled brain subdivisions within the customized atlas, which were generated using FreeSurfer-based structural image parcellation, were registered to the diffusion space with the transformation matrix from structural to diffusion space generated by FSL FDT registration process^4^. The result of this step was a brain parcellation that was defined in the diffusion space.

Then, we conducted probabilistic fiber tacking (Probtrackx in FSL) (Behrens et al., 2007) to delineate connections between the thalamus and all of the subdivisions in the ipsilateral hemisphere. For probabilistic fiber tracking, we set the streamline number for each seed voxel to 5,000, curvature threshold to 0.2, maximum number of steps to 2,000, and a step length of 0.5 mm. The thalamus was set as the seed mask, and another brain subdivision was set as the waypoint mask and the termination mask. A threshold of 100 was used for the connectivity map to avoid spurious results as the consequence of image noise (Ciccarelli et al., 2006; Rajagopal et al., 2013). For the subdivision pair (A, B), if the tract between A and B passed a voxel, then the streamline number of that voxel was non-zero. A tract mask was the binary mask in which a voxel value was 1 if the voxel had a non-zero streamline number. For the subdivision pair (A, B), we calculated the following features: the CS, which was the average streamline number of voxels inside the tract mask and indicated the strength of structural connections between the distinct regions, and the average diffusion metric value of voxels inside the tract mask. The diffusion metrics included fractional anisotropy (FA), mean diffusivity (MD), axial diffusivity (AD), and radial diffusivity (RD). FA measures the degree of anisotropy of water molecules, which could reflect the white matter microstructural integrity; MD is a measure of molecular motion averaged over all directions and could be associated with white matter edema; and AD and RD represent the diffusivities parallel and perpendicular to the fiber, which could reflect axonal and myelin integrity, respectively (Keiding and Pavese, 2013).

The one-way analysis of variance was conducted to examine the difference in the above features across the three groups. The false discovery rate (FDR)-corrected *P* value < 0.05 was deemed to be statistically significant.

### Correlation Analysis

For the metrics with significant differences during between-group comparisons, we extracted their values and correlated these with the cirrhotic patients’ cognition performance (indexed by PHES). Then, Pearson correlation analysis was performed, and an FDR-corrected *P* value < 0.05 was deemed to be statistically significant.

### Classification Analysis

For the classification task, we constructed a multilayer perceptron (MLP) model that had one hidden layer with 256 nodes. We chose this neural network architecture because we had a limited number of samples and a model with many hidden layers could overfit. The predictors were the metrics with significant differences during between-group comparisons. These predictors were normalized to zero mean and unit variance. The outcome variable was with and without MHE (a binary variable). Our implementation was based on Tensorflow. We used the Adam algorithm to train the neural network with sparse categorical cross entropy as the loss function and epochs = 100. We use 10-fold cross-validation to assess model performance. In *k*-fold cross-validation, the original dataset is divided into *k* subsamples. Then, we trained the model on *k*-1 subsamples and tested the model on the left-out subsample. We repeated this process *k* times and calculated the average performance. Cross-validation provides a reliable assessment of model performance because the model never uses any case from the test dataset. Model quality metrics for classification consisted of accuracy, sensitivity, and specificity.

## Results

**FIGURE 2:**
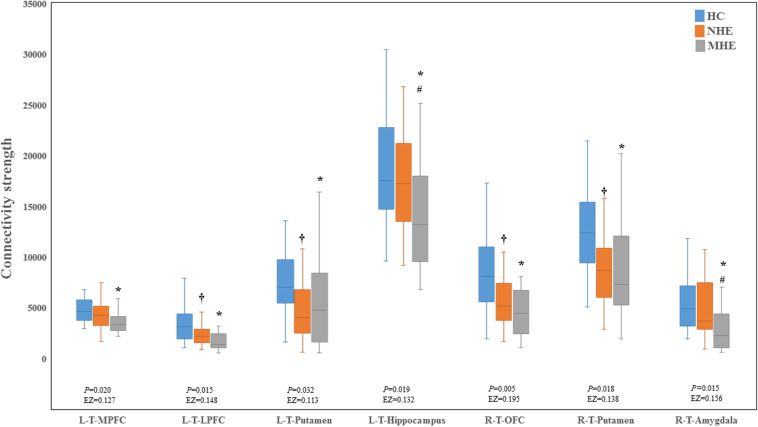
Between-group differences in connectivity strength. The false discovery rate (FDR)-corrected *P* values and the effect size (EZ) are shown. The symbols *, ^†^, and ^#^ indicate significant differences in MHE vs. HC, NHE vs. HC, and MHE vs. NHE, respectively. MHE, minimal hepatic encephalopathy; NHE, patients without MHE; HC, healthy control; LPFC, lateral prefrontal cortex; MPFC, medial prefrontal cortex; OFC, orbitofrontal cortex; T, thalamus; L, left; R, right.

**FIGURE 3:**
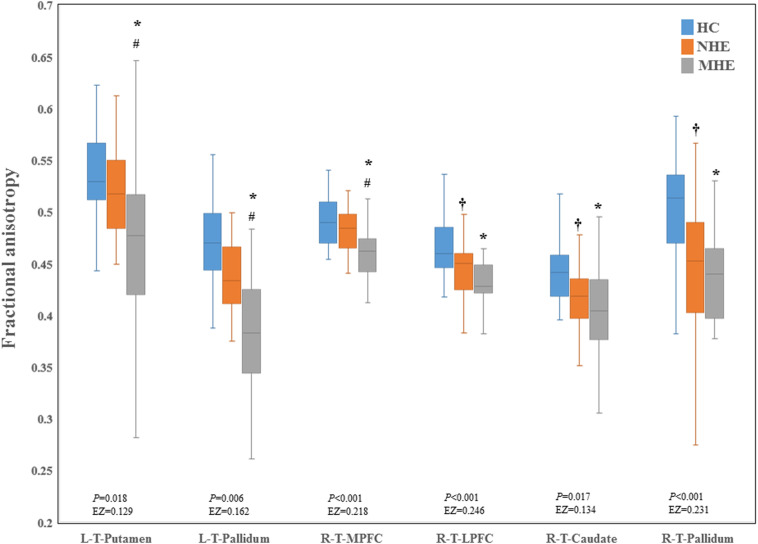
Between-group difference in fractional anisotropy. The false discovery rate (FDR)-corrected *P* values and the effect size (EZ) are shown. The symbols *, ^†^, and ^#^ indicate significant differences in MHE vs. HC, NHE vs. HC, and MHE vs. NHE, respectively. MHE, minimal hepatic encephalopathy; NHE, patients without MHE; HC, healthy control; LPFC, lateral prefrontal cortex; MPFC, medial prefrontal cortex; T, thalamus; L, left; R, right.

**FIGURE 4:**
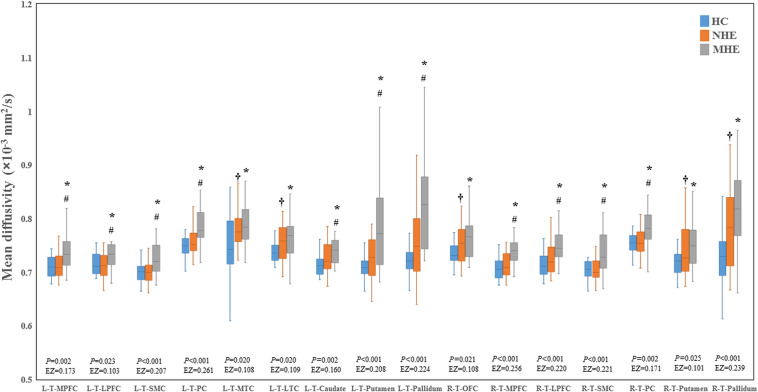
Between-group differences in mean diffusivity. The false discovery rate (FDR)-corrected *P* values and the effect size (EZ) are shown. The symbols *, ^†^, and ^#^ indicate significant differences in MHE vs. HC, NHE vs. HC, and MHE vs. NHE, respectively. MHE, minimal hepatic encephalopathy; NHE, patients without MHE; HC, healthy control; LPFC, lateral prefrontal cortex; LTC, lateral temporal cortex; MPFC, medial prefrontal cortex; MTC, medial temporal cortex; OFC, orbitofrontal cortex; PC, parietal cortex; SMC, sensorimotor cortex; T, thalamus; L, left; R, right.

**FIGURE 5:**
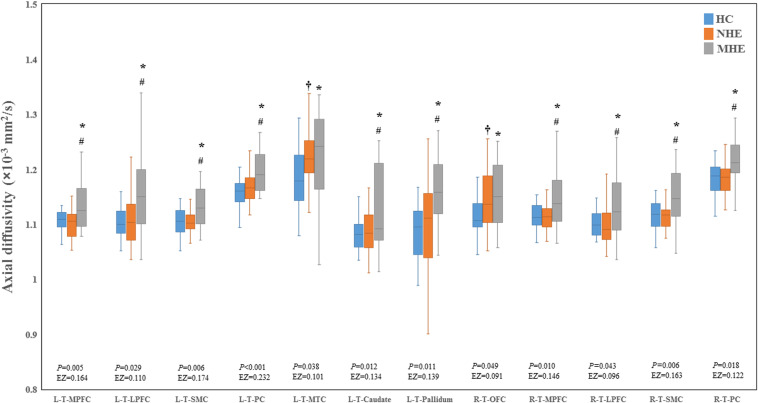
Between-group differences in axial diffusivity. The false discovery rate (FDR)-corrected *P* values and the effect size (EZ) are shown. The symbols *, ^†^, and ^#^ indicate significant differences in MHE vs. HC, NHE vs. HC, and MHE vs. NHE, respectively. MHE, minimal hepatic encephalopathy; NHE, patients without MHE; HC, healthy control; LPFC, lateral prefrontal cortex; MPFC, medial prefrontal cortex; MTC, medial temporal cortex; OFC, orbitofrontal cortex; PC, parietal cortex; SMC, sensorimotor cortex; T, thalamus; L, left; R, right.

**FIGURE 6:**
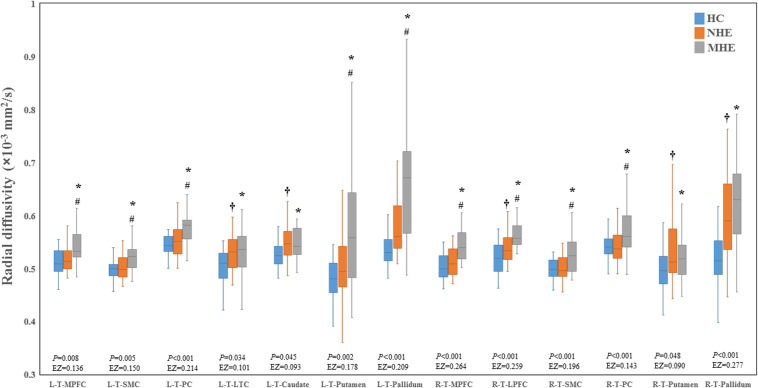
Between-group differences in radial diffusivity. The false discovery rate (FDR)-corrected *P* values and the effect size (EZ) are shown. The symbols *, ^†^, and ^#^ indicate significant differences in MHE vs. HC, NHE vs. HC, and MHE vs. NHE, respectively. MHE, minimal hepatic encephalopathy; NHE, patients without MHE; HC, healthy control; LPFC, lateral prefrontal cortex; LTC, lateral temporal cortex; MPFC, medial prefrontal cortex; PC, parietal cortex; SMC, sensorimotor cortex; T, thalamus; L, left; R, right.

Figure 2 shows the CS differences across the three groups. Compared with HC, the MHE patients exhibited a decrease in CS in several fibers, including L (Left)-T (Thalamus)-MPFC, L-T-LPFC, L-T-Putamen, L-T-Hippocampus, R (Right)-T-OFC, R-T-Putamen, and R-T-Amygdala, which indicated the reduction of interregional structural connectivity. Meanwhile, compared with HC, MHE patients showed decreased FA along the following fiber, including L-T-Putamen, L-T-Pallidum, R-T-MPFC, R-T-LPFC, R-T-Caudate, and R-T-Pallidum (Figure 3), which indicated the impaired microstructural integrity of fibers. Figure 4 shows the increased MD in MHE patients compared with that in HC subjects, which involved a set of fibers, including L-T-MPFC, L-T-LPFC, L-T-SMC, L-T-PC, L-T-MTC, L-T-LTC, L-T-Caudate, L-T-Putamen, L-T-Pallidum, R-T-OFC, R-T-MPFC, R-T-LPFC, R-T-SMC, R-T-PC, R-T-Putamen, and R-T-Pallidum. The increase in MD may be associated with the edema along white matter pathways that commonly occurs in cirrhosis (Singhal et al., 2010; Keiding and Pavese, 2013). In addition, Figures 5, 6 show the AD and RD increments in MHE. Most of the fibers with increased AD or RD also showed the increment in MD. The increased AD and RD could reflect the axonal and myelin injury in MHE, respectively. Notably, a stepwise change of CS, FA, MD, AD, and RD was observed from the NHE to the MHE group, indicating the potential of these measures for monitoring disease progression.

Significantly positive correlations were observed between the PHES result (that indicated the cognitive dysfunction in cirrhotic patients) and CS values along L-T-LPFC, L-T-Hippocampus, and R-T-Amygdala (Table 2). The positive correlations were also observed between PHES result and FA values along the L-T-Putamen, L-T-Pallidum, R-T-MPFC, and R-T-LPFC. In addition, we observed significant negative correlations between the PHES results and MD values in the following connections: bilateral T-MPFC, bilateral T-LPFC, bilateral T-SMC, bilateral T-PC, L-T-Caudate, L-T-Putamen, and bilateral T-Pallidum. The results of correlation analyses based on AD and RD were similar to those based on MD metrics (Table 2).

**TABLE 2:** Correlations between thalamic connectivity measurements and the PHES.

Connection	CS		FA		MD		AD		RD	
	CC	*P* value	CC	*P* value	CC	*P* value	CC	*P* value	CC	*P* value
L-T-MPFC	0.168	0.137			−0.450	**0.001**	−0.468	**0.001**	−0.386	**0.005**
L-T-LPFC	0.361	**0.012**			−0.319	**0.015**	−0.250	**0.044**		
L-T-SMC					−0.468	**0.001**	−0.511	**0.001**	−0.369	**0.006**
L-T-PC					−0.449	**0.001**	−0.432	**0.002**	−0.409	**0.005**
L-T-MTC					−0.031	0.415	−0.027	0.424		
L-T-LTC					−0.107	0.241			−0.038	0.394
L-T-Caudate					−0.305	**0.018**	−0.389	**0.004**	−0.099	0.287
L-T-Putamen	0.175	0.137	0.334	**0.016**	−0.449	**0.001**			−0.408	**0.005**
L-T-Pallidum			0.366	**0.011**	−0.408	**0.003**	−0.306	**0.018**	−0.397	**0.005**
L-T-Hippocampus	0.348	**0.020**								
R-T-OFC	0.199	0.137			−0.138	0.188	−0.067	0.346		
R-T-MPFC			0.388	**0.011**	−0.439	**0.002**	−0.389	**0.004**	−0.428	**0.005**
R-T-LPFC			0.302	**0.022**	−0.369	**0.006**	−0.313	**0.018**	−0.366	**0.006**
R-T-SMC					−0.458	**0.001**	−0.463	**0.001**	−0.403	**0.005**
R-T-PC					−0.397	**0.004**	−0.350	**0.009**	−0.369	**0.006**
R-T-Putamen	0.102	0.235			−0.233	0.060			−0.078	0.317
R-T-Caudate			0.217	0.073						
R-T-Pallidum			0.130	0.179	−0.348	**0.009**			−0.288	**0.025**
R-T-Amygdala	0.406	**0.010**								

*Significant correlations (with FDR-corrected P value < 0.05) are indicated in bold. PHES, Psychometric Hepatic Encephalopathy Score; CS, connectivity strength; FA, fractional anisotropy; MD, mean diffusivity; AD, axial diffusivity; RD, radial diffusivity; CC, correlation coefficient; L, left; R, right; LPFC, lateral prefrontal cortex; LTC, lateral temporal cortex; MPFC, medial prefrontal cortex; MTC, medial temporal cortex; OFC, orbitofrontal cortex; PC, parietal cortex; SMC, sensorimotor cortex.*

The classification results are summarized in Table 3. The relatively high accuracy of 0.807 (with sensitivity = 0.900 and specificity = 0.681) was obtained using the CS index, whereas the moderate accuracy of 0.711 (with sensitivity = 0.700 and specificity = 0.727) was obtained using the MD index. The application of the FA, AD, and RD metrics did not yield good classification results.

**TABLE 3:** The performance of classification analysis between the cirrhotic patients with and without MHE.

Predictors	Accuracy	Sensitivity	Specificity	Features contributing most to classification (ranked by the discrimination weight)
CS	0.807	0.900	0.681	CS value along 7 connections: R-T-Amygdala, L-T-Hippocampus, L-T-LPFC, L-T-MPFC, R-T-OFC, R-T-Putamen, and L-T-Putamen
FA	0.558	0.600	0.500	FA value along 6 connections: R-T-MPFC, L-T-Pallidum, L-T-Putamen, R-T- Caudate, R-T-LPFC, and R-T-Pallidum
MD	0.711	0.700	0.727	MD value along 16 connections: L-T-Pallidum, L-T-PC, L-T-SMC, R-T-MPFC, R-T-SMC, L-T-Putamen, R-T-LPFC, L-T-MPFC, R-T-PC, L-T-LPFC, L-T-LTC, L-T-Caudate, L-T-MTC, R-T-Pallidum, R-T-OFC, and R-T-Putamen
AD	0.577	0.700	0.409	AD value along 12 connections: L-T-SMC, R-T-PC, L-T-MTC, L-T-Pallidum, L-T-LPFC, R-T-SMC, R-T-MPFC, L-T-MPFC, L-T-Caudate, L-T-PC, R-T-LPFC, and R-T-OFC
RD	0.634	0.700	0.545	RD value along 13 connections: L-T-Putamen, R-T-LPFC, L-T-PC, R-T-MPFC, L-T-Pallidum, L-T-MPFC, R-T-SMC, L-T-LTC, R-T-PC, L-T-SMC, L-T-Caudate, R-T-Putamen, and R-T-Pallidum

*MHE, minimal hepatic encephalopathy; CS, connectivity strength; FA, fractional anisotropy; MD, mean diffusivity; AD, axial diffusivity; RD, radial diffusivity; L, left; R, right; LPFC, lateral prefrontal cortex; LTC, lateral temporal cortex; MPFC, medial prefrontal cortex; MTC, medial temporal cortex; OFC, orbitofrontal cortex; PC, parietal cortex; SMC, sensorimotor cortex.*

## Discussion

Using diffusion-based probabilistic tractography analysis, we revealed altered thalamic structural connectivity in MHE primarily involving the prefrontal cortex, SMC, PC, MTC, hippocampus, and striatum. Our primary results were as follows: (1) the disruption of thalamic connectivity in cirrhotic patients, as reflected by a reduction in FA and CS and an increase in MD, AD, and RD; (2) a progressively disturbed thalamic connection from NHE to MHE, which supports the idea that HE is a continuum of neurological dysfunction (Bajaj et al., 2009), and thus the aberrant thalamic connection may be used to dynamically monitor the development of disease; (3) the correlation between thalamic structural connectivity and patients’ cognitive performance, which suggested that the disturbed thalamic connectivity may be the critical neurobiological basis of MHE; and (4) the high classification accuracy, which suggested that thalamic structural connectivity alterations can effectively distinguish MHE from NHE.

A reduction in FA coupled with an increase in MD, AD, and RD is generally interpreted as the injury involving both axon and myelin (Madden et al., 2012). Loss of axons (Matsusue et al., 2005) and impaired axonal integrity (indicated by decreased axial kurtosis) (Chen et al., 2017) have been noted in cirrhotic patients. In MHE, astrocyte dysfunction has been well documented (Chen and Herskovits, 2005), which may further result in axonal degeneration as reported in multiple sclerosis (Cambron et al., 2012). In addition, the demyelination has been shown to play a major role in the pathogenesis of MHE. The oxidative injury and inflammation have been observed in MHE (Atluri et al., 2011), both of which may lead to oligodendrocyte death and, subsequently, demyelination (McTigue and Tripathi, 2008). Taken together, a reduction in FA combined with increased MD, AD, and RD may suggest impaired white matter microstructure in MHE and may contribute to CS alterations. Moreover, the finding of decreased thalamic CS is consistent with that of previous studies, revealing decreased thalamic functional connectivity in MHE (Qi et al., 2013).

Our result of disturbed thalamo-prefrontal structural connectivity coincided with the findings of previous neuroimaging investigations demonstrating microstructural abnormality in the anterior thalamic radiation (a connective structure between the prefrontal cortex and the thalamus) due to MHE (Montoliu et al., 2014; Li et al., 2019). Other investigations that generated results that were concordant with our findings conducted functional connectivity analyses, which revealed less connectivity between the prefrontal cortex and thalamus (Qi et al., 2013; Chen et al., 2015). In fact, three distinct prefrontal–subcortical (including the thalamus) circuits and their associated neurobehavioral syndromes have been recognized (Tessitore et al., 2012): the abnormalities in the circuit between the dorsolateral prefrontal and thalamus can correspond to impairments in executive function and motor programming (Cummings, 1993); the dysfunction in medial frontal–anterior cingulate circuit (primarily composed of the anterior cingulate cortex, striatal, and thalamus) is associated with personality changes such as apathy and diminished initiative (Cummings, 1993); and disinhibition and irritability indicate a disturbance of the orbitofrontal pathway consisting of the orbitofrontal cortex, striatal, subthalamic nucleus, and ventral anterior and mediodorsal thalamic nuclei (Cummings, 1993). Of note, the executive dysfunction, impaired set shifting and maintains, abnormal motor programming, apathy, dysphoria, fretfulness, and irritability have been observed in MHE/HE (Vilstrup et al., 2014). Thus, we inferred that the abnormal thalamo–prefrontal connection may account for the pathobiological bases of these neuropsychological symptoms.

In line with our finding of disrupted thalamo-SMC connectivity in MHE, functional analyses have demonstrated decreased thalamic functional connectivity with motor-related regions (Qi et al., 2013; Chen et al., 2015). Previous studies have indicated that white matter connectivity between the thalamus and motor-associated region is associated with motor task performance (Xia et al., 2012; Philp et al., 2014). Consistently, a previous magnetoencephalography study revealed a relationship between altered thalamo-motor-cortical coupling and motor dysfunction in MHE (Timmermann et al., 2003). Therefore, it is possible that the impaired thalamo-SMC connectivity is associated with motor dysfunction in MHE, such as impaired bimanual coordination, bradykinesia, movement initiation, and inhibition dysfunction (Giewekemeyer et al., 2007; Vilstrup et al., 2014).

Deficits in spatial cognition, such as spatial orientation, spatial attention shift, and visuospatial reasoning, are important characteristics of MHE (Vilstrup et al., 2014). The present study revealed disturbed structural connectivity between the thalamus and PC (a region responsible for spatial cognitive process) in MHE. In fact, our observation of disrupted anatomical connectivity involving PC is concordant with a previous functional study that revealed decreased functional connectivity between the PC and thalamus (Zhang et al., 2012). Given that the function of specific cortical areas is constrained and determined by their anatomical connections (Klein et al., 2010), we hypothesize that the perturbed thalamic connection with PC may affect PC-related spatial cognition function, leading to the relevant cognitive impairments in MHE.

The impaired thalamic connectivity with the hippocampus and MTC agrees with the findings of previous research. For example, analysis of hippocampal connectivity showed a stepwise reduction in functional connectivity between the hippocampus and thalamus during progression from NHE to MHE (Lin et al., 2019). Furthermore, the disruption in functional connectivity between the MTC and thalamus has been observed in cirrhotic patients without OHE (Zhang et al., 2012; Chen et al., 2015). The theories of dual/multiple systems involving memory wherein multiple neural circuits connect the anterior and mediodorsal thalamic nuclei to the hippocampus and rhinal/parahippocampal cortices well support memory function (Nishio et al., 2014). Moreover, a previous study has demonstrated the correlation between disrupted memory system and memory deficits in patients with amnestic mild cognitive impairment (Brueggen et al., 2016). Thus, the disruption of thalamic connectivity between the MTC and hippocampus may also be responsible for the observed memory deficits in MHE.

Our finding of disturbed structural connectivity between the thalamus and striatum is concordant with results of previous functional connectivity analyses that revealed impaired thalamic–striatal functional interaction in cirrhotic patients without OHE (Qi et al., 2013; Chen et al., 2015). Functionally, the thalamic–striatal system subserves attention shifting, alertness regulation, and behavioral switching (Smith et al., 2011), all of which were impaired in MHE (Prasad et al., 2007; Bajaj et al., 2009). Previous studies have suggested that degeneration of the thalamic–striatal system may contribute to set shifts and behavioral switching deficits (Smith et al., 2011). Thus, we hypothesize that the impaired thalamic–striatal system is associated with relevant cognitive deficits in MHE.

First, the statistic power of our results is limited by the small sample size, and further studies involving more participants are encouraged. Second, as a cross-sectional study, the inference about progressive thalamic connectivity degeneration in the spectrum of HE is speculative. A longitudinal study is needed to corroborate this speculation. Third, the thalamus is a complex structure composed of several subregions with different connections and functions. Thus, additional studies evaluating the MHE-related alterations in the connection between these specific thalamic nuclei and cortical/subcortical areas are warranted. Fourth, as a single tensor model, the DTI-based tractography is unable to fully account for crossing or branching fibers, which may cause inaccurate tractography inevitably, to some extent. Another study applying more advanced MR sequence is warranted to alleviate this problem. Fifth, the current study only evaluated the thalamic–cortical/subcortical connections in the cirrhotic patient with MHE; however, previous studies have implied that the cirrhosis could result in widespread white matter structural impairments (Guevara et al., 2011; Lin et al., 2012; Chen et al., 2017). Thus, our finding of altered thalamic connections should be regarded as representative of MHE-related widespread impairments in white matter pathways. In addition to thalamic–cortical/subcortical connections, further studies are recommended to explore the structural alterations in other white matter pathways in MHE.

Our results demonstrate the altered thalamic structural connectivity with both cortical and subcortical regions in MHE. The disruption in thalamic connectivity may be the underlying mechanism of cognitive deficits in MHE and may be potentially utilized as a biomarker for MHE diagnosis and in monitoring disease progression.

## Data Availability Statement

The original contributions presented in the study are included in the article/supplementary material, further inquiries can be directed to the corresponding authors.

## Ethics Statement

The studies involving human participants were reviewed and approved by the Research Ethics Committee of Fujian Medical University Union Hospital and The First Affiliated Hospital of Nanjing Medical University. The patients/participants provided their written informed consent to participate in this study.

## Author Contributions

H-JC, X-HZ, DL, and RC conceived and designed the study, acquired and analyzed the data, and wrote the manuscript. J-YS, S-FJ, Y-FS, and LZ acquired and analyzed the data. All authors have read and approved the manuscript.

## Conflict of Interest

The authors declare that the research was conducted in the absence of any commercial or financial relationships that could be construed as a potential conflict of interest.

## Footnotes

1 http://www.freesurfer.net/

2 https://surfer.nmr.mgh.harvard.edu/fswiki/FsTutorial/AnatomicalROI

3 https://fsl.fmrib.ox.ac.uk/fsl/fslwiki/

4 https://fsl.fmrib.ox.ac.uk/fsl/fslwiki/FDT

## Abbreviations

AD: axial diffusivity
CS: connectivity strength
DTI: diffusion tensor imaging
FA: fractional anisotropy
HCs: healthy controls
HE: hepatic encephalopathy
LPFC: lateral prefrontal cortex
LTC: lateral temporal cortex
MD: mean diffusivity
MHE: minimal hepatic encephalopathy
MPFC: medial prefrontal cortex
MTC: medial temporal cortex
NHE: cirrhotic patients without MHE
OCC: occipital cortex
OFC: orbitofrontal cortex
OHE: overt hepatic encephalopathy
PC: parietal cortex
PHES: Psychometric Hepatic Encephalopathy Score
RD: radial diffusivity
SMC: sensorimotor cortex.
